# Early-Onset Cone Photoreceptor Degeneration Is Associated With High Myopia in *RPGR*-Related Retinal Dystrophy

**DOI:** 10.1155/joph/4244740

**Published:** 2025-06-11

**Authors:** Shabnam Raji, Laura J. Taylor, Amandeep S. Josan, Robert E. MacLaren, Jasmina Cehajic-Kapetanovic

**Affiliations:** ^1^Oxford Eye Hospital, Oxford University Hospitals NHS Foundation Trust, Oxford, UK; ^2^Nuffield Laboratory of Ophthalmology, Department of Clinical Neurosciences, University of Oxford, Oxford, UK

## Abstract

**Purpose:** High myopia is a feature of several inherited retinal diseases, including X-linked retinitis pigmentosa (XLRP) which is characterized by childhood onset, centripetal photoreceptor degeneration, and rapid progression to blindness by the fourth decade. Mutations in the retinitis pigmentosa GTPase regulator (RPGR) gene cause over 90% of XLRP cases. It presents with a varied clinical phenotype, categorized into the predominant rod-cone, cone-rod, and cone dystrophy. This case-series study examines the clinical characteristics of patients with *RPGR*-related retinal dystrophy to identify associations with refractive error.

**Methods:** Data collected between October 2023 and April 2024 from retinal imaging, clinical ophthalmic examination, and genetic analysis were retrospectively analyzed.

**Results:** Twenty-four male patients were identified, with a mean age of 30 years (range 7–57). The median (IQR) best-corrected visual acuity was 60 (55–66) letters in the cone-rod/cone phenotype and 65 (49–73) letters in the rod-cone phenotype. High axial myopia showed preponderance in cone-dominated degenerations. Estimated mean refractive error was −7.92DS (95% CI: [−11.39, −4.44]) in the cone-rod phenotype and −3.52DS (95% CI: [−5.87, −1.17]) in the rod-cone phenotype, adjusting for age and genetic mutation. This difference between phenotype was significant (*p*=0.041). In a subanalysis, no significant association was found between refractive error and nucleotide position. Evaluation of disease progression found that all patients with a fast-progressing, rod-cone phenotype had high myopia. Conversely, one patient who presented with a slow-progressing, cone-rod phenotype did not have high myopia.

**Conclusions:** Refractive trends in this cohort suggest that cone photoreceptor degeneration occurring during early childhood is associated with high myopia. Image degradation primarily due to cone photoreceptor dysfunction may act as a stimulus to drive myopia development in early childhood. These observations advocate for the earlier treatment of myopia in cone-dominated *RPGR*-related retinal dystrophy to preserve retinal function and minimize the risks of retinal gene therapy surgery for patients enrolling in clinical trials.

**Trial Registration:** ClinicalTrials.gov identifier: NCT03116113

## 1. Introduction

Myopia is a refractive error in which light focuses in front of the retina when the eye is in a nonaccommodative state. It is usually caused by elongation of the globe with high myopia classified by an axial length of > 26 mm or dioptrically as > −6.00 D [1]. Although most symptoms of myopia can be relieved by spectacles, contact lenses, or refractive surgery, structural changes to the retina arising from pathologic myopia pose a high risk of irreversible visual impairment and can lead to blindness [2]. Early-onset myopia occurs before the age of around 10–13 years and develops when normal eye growth is still occurring [1]. It is associated with a higher rate of myopia progression and severity, and is more likely to have a genetic origin [3]. Importantly, the onset of myopia often affects a person's productive years creating a significant social and economic burden on society [4]. The exact mechanisms of myopia development remain largely unknown, although a multifactorial etiology is well accepted, composed of environmental and genetic risk factors.

High myopia is a feature of several inherited retinal diseases, including retinitis pigmentosa GTPase regulator (*RPGR*)-related retinal dystrophy which has among the highest level of myopic refractive error [5]. Mutations in *RPGR* cause over 90% of X-linked retinitis pigmentosa (XLRP) cases which is characterized by childhood onset, centripetal photoreceptor degeneration and rapid progression to blindness by the fourth decade when cones are affected later in the disease. *RPGR*-related retinal dystrophy presents phenotypic variability, with either cone or rod dominated degeneration, and can be categorized into rod-cone (encompassing XLRP), cone-rod, or cone dystrophy phenotype. The cone-rod phenotype, also referred to as X-linked cone-rod dystrophy (CRD), affects cones then rods, giving rise to a progressive decline in visual acuity and dyschromatopsia, followed by nyctalopia [6]. It has been found that distal truncating mutations occurring toward the open-reading frame 15 (ORF15) of the *RPGR*^*ORF*15^ isoform result in a shift toward the cone-dominated phenotype, while mutations in exons 1–14 associate with the rod-dominated phenotype [7]. *RPGR*-related retinal dystrophy is currently an untreatable condition, although clinical trials investigating novel gene therapies are underway [8, 9]—the co-occurrence of pathological myopia with retinal dystrophy can lead to a guarded visual prognosis. Several studies have reported the presence of myopia or increased axial length in *RPGR*-related retinal dystrophies, specifically in patients with pathogenic variants in the *RPGR*^ORF15^ region, which is associated with a faster decline in best-corrected visual acuity (BCVA) due to earlier macula involvement [5, 10–15].

The aim of this study is to present the clinical characteristics of 24 patients with molecularly confirmed *RPGR*-related retinal dystrophy in order to identify associations with refractive error and better understand the dominance of myopic refractive error in this patient cohort. These observations may have implications for patient selection in clinical trials but, more broadly, may allude to possible mechanisms of myopia development.

## 2. Methods

### 2.1. Study Design

The study design adhered to the tenets of the Declaration of Helsinki [16]. The study followed the reporting guidelines for case series. Patients were retrospectively identified from prior enrollment in a clinical trial for *RPGR*-related XLRP and from specialist retina and genetics clinics at the Oxford Eye Hospital, Oxford, United Kingdom. Informed written consent was obtained from patients prior to their enrollment into the clinical trial.

### 2.2. Inclusion and Exclusion Criteria

The criteria for inclusion into the present study were a genetically confirmed mutation in the *RPGR gene*, phakia, recorded refraction and an absent history of refractive surgery or other procedures known to affect refractive error. Carriers of *RPGR* mutations were excluded. No restriction was placed on age or ethnicity.

### 2.3. Clinical Data Collection

Data from patient records between October 2023 and April 2024 was retrospectively reviewed and analyzed. All data was collected from the screening visit prior to any interventional treatment. Medical, ophthalmic, and family history were collected that included self-reported onset of spectacle use and visual symptoms. All patients had retinal imaging to include color fundus photography, fundus autofluorescence (55° and 30°), and spectral-domain optical coherence tomography captured by the Spectralis (Heidelberg Engineering GmbH, Heidelberg, Germany). Full-field electroretinography was measured using Espion (Diagnosys LLC, Lowell, MA, USA) following the International Society for Clinical Electrophysiology of Vision (ISCEV) standard protocol. Genetic analysis was performed by targeted next-generation sequencing techniques and panel-based sequencing of all inherited retinal disease genes with pathogenic variants confirmed by Sanger sequencing. A Manifest refraction was collected, and the spherical equivalent refractive error was used for subsequent analysis, calculated to three decimal places for each eye by the sphere power plus half of the cylinder power. BCVA and refractive error was measured to the standardized Early Treatment for Diabetic Retinopathy Study (ETDRS) protocol. Axial length measurements were obtained using the Zeiss IOLMaster 700 (Carl Zeiss Meditec AG, Jena, Germany).

Patients were classified into a rod-cone, cone-rod, or cone-only phenotype based on genetic testing and clinical phenotyping using multimodal retinal imaging, clinical history, and electrodiagnostic testing. A rod-cone phenotype was determined by a clinical history of nyctalopia and visual field restriction, and the presence of bone spicule pigmentation or peripheral atrophy on wide-field retinal imaging. This clinical presentation was reflected by full-field electroretinography which showed greater dysfunction in rods compared to cones. A cone-rod phenotype was determined by a clinical history of blurred vision, visual issues in photopic light, photophobia, and dyschromatopsia. Patients with a cone-rod phenotype present with an earlier onset and more pronounced cone-related degeneration compared to rod-related degeneration. Retinal imaging features include a hyperautofluorescent ring encircling an area of hypo-autofluorescence and attenuated ellipsoid zone on optical coherence tomography. Full-field electroretinography typically showed greater dysfunction in cones than rods. The cone-only phenotype presented similarly to the cone-rod phenotype, but with no peripheral pigmentary retinopathy or rod-related degeneration.

### 2.4. Statistical Analysis

Descriptive statistics were used to summarize clinical data, with counts and percentages reported for categorical variables, and medians and interquartile ranges (IQR) calculated for continuous variables. Simple linear regression was used to determine the interocular symmetry in refractive error, and the results were reported as the square of Pearson's correlation coefficient. The correlation between spherical equivalent refractive error and axial length was determined using a linear mixed model. Multivariate linear mixed model regression analysis was used to evaluate the association between phenotype and refractive error. Phenotype (cone-rod or rod-cone) was set as the predictor variable, and refractive error was the outcome variable. The model adjusted for age and location of genetic mutation (ORF15 or exon 1–14) as fixed effects and patient ID as a random intercept to account for the nonindependence of right and left eye data from each patient. The null hypothesis that there is no difference in the estimated mean refractive error of each phenotype was tested using a *t*-test. A *p*-value of less than 0.05 was considered statistically significant. To clarify the independent contribution of mutation location to refractive error, univariate regression subanalysis was performed to evaluate the association between phenotype and refractive error within the ORF15 region only. Further regression analysis was performed to evaluate the correlation between nucleotide position and refractive error. Statistical analysis was conducted in R (version 4.1.2; R Foundation for Statistical Computing, Vienna, Austria). Diagrams were produced in Inkscape (version 0.92).

## 3. Results

Twenty-four patients were identified according to inclusion and exclusion criteria. The demographic characteristics and clinical information of all included patients is shown in Table 1. All patients were White males, except for one Asian male. The age at the time of refraction ranged from 7 to 57 years with a mean age of 30 years. Eight (33%) patients were identified as having a cone-rod phenotype, one (4%) with a cone-only phenotype, and the remaining 15 (62%) patients had a rod-cone phenotype.

The median (IQR) BCVA was 60 (55–66) letters in the cone-rod/cone-only phenotype and 65 (49–73) letters in the rod-cone phenotype. The median (IQR) spherical equivalent refractive error was −7.63DS (−10.25 to −4.00) in the cone-rod/cone-only phenotype and −3.00DS (−4.50 to −0.19) in the rod-cone phenotype.

The right and left eye refractive error were significantly correlated, and as such, this cohort exhibited a high degree of interocular symmetry in refractive error (*r*^2^ = 0.90, *p* < 0.001). A significant negative correlation was found between spherical equivalent refractive error and axial length, indicating axial myopia in this cohort (*R*^2^ = 0.99, *p* < 0.001). Axial length measurements were not available in 10 patients.

**Figure 1:**
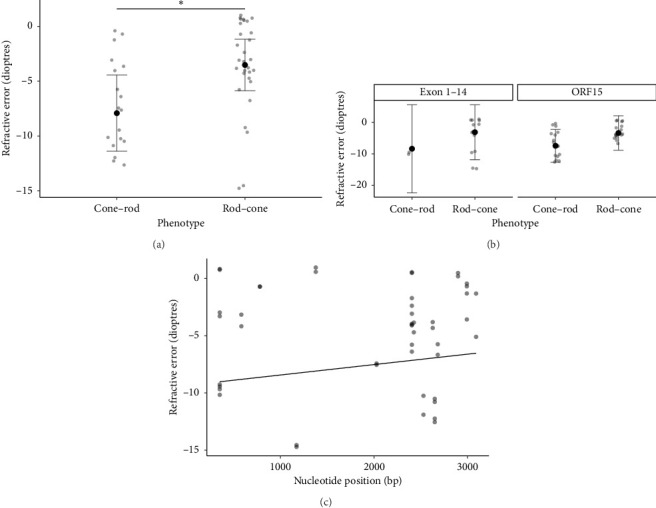
Linear regression analysis showing (a) the predicted spherical equivalent refractive error by phenotype in *RPGR*-related retinal dystrophy. The estimated mean refractive error was significantly more myopic in the cone-rod phenotype (−7.92DS, 95% CI: [−11.39, −4.44]) compared to the rod-cone phenotype (−3.52DS, 95% CI: [−5.87, −1.17]). (b) Predicted spherical equivalent refractive error by phenotype within the ORF15 and exon 1–14 region of *RPGR*^*ORF*15^. When isolating the analysis to only ORF15, the predicted mean refractive error remained more myopic in the cone-rod phenotype (−6.89DS, 95% CI: [−10.22, −3.57]) compared to the rod-cone phenotype (−2.77DS, 95% CI: [−6.09, 0.54]), although this difference was not significant (*p*=0.08). (c) No significant correlation was found in a subanalysis of predicted spherical equivalent refractive error by nucleotide position (*β* = 0.0009, 95% CI: [−0.001, 0.0028], *p*=0.346). All data points show the distribution of actual spherical equivalent refractive error from the right and left eye of all patients. An asterisk indicates *p* < 0.05.

Relative to the −6.00D threshold of high myopia, higher levels of myopic refractive error showed preponderance in patients with cone-dominated degenerations, as shown in Figure 1. Linear mixed model regression analysis predicted an estimated mean refractive error of −7.92DS (95% CI: [−11.39, −4.44]) in the cone-rod phenotype and −3.52DS (95% CI: [−5.87, −1.17]) in the rod-cone phenotype. The estimated mean refractive error was significantly different between phenotype (*p*=0.041). The narrower confidence intervals in the rod-cone phenotype indicated better precision of the estimated mean compared to the cone-rod phenotype.  Figure 1(b) presents the results of univariate regression analysis conducted on patients harboring only ORF15 mutations. This analysis found that those with a cone-rod phenotype were more myopic compared to rod-cone (−6.89DS, 95% CI: [−10.22, −3.57] in cone-rod vs. −2.77DS, 95% CI: [−6.09, 0.54] in rod-cone, [*p*=0.08]). It should be noted that the subanalysis in Figure 1(b) may also be underpowered to detect statistically significant associations due to the smaller sample size. Further regression analysis shown in Figure 1(c) demonstrated no significant correlation between nucleotide position and refractive error (*β* = 0.0009, 95% CI: [−0.001, 0.0028], *p*=0.346). Collectively, the sub-analyses in Figures 1(b) and 1(c) suggest that genetic mutation does not have a significant independent contribution to refractive error; variations in refractive error are more likely attributable to disease phenotype. Evidence of pathologic myopia featured in this cohort of patients include a posterior staphyloma in patient 17, and retinal detachment in patient 21.

In 23 out of 24 (96%) patients, the astigmatism was meridionally symmetrical. All patients had a cylinder of less than −2.50DC except patients 23 and 24 who exhibited symmetrical oblique astigmatism of −4.25DC and −3.00DC, respectively; the high cylinder power could suggest a heritable component to this astigmatism, but this could not be confirmed due to an absent family history of refractive error.

The commonest self-reported symptom indicative of retinal dystrophy was nyctalopia (14/24, 58%) followed by visual field restriction (4/24, 16%) and dyschromatopsia (1/24, 4%), the last only reported in a patient with a cone-rod phenotype. The median self-reported age at onset of nyctalopia symptoms was 20 years in the cone-rod phenotype and 8.5 years in the rod-cone phenotype. The self-reported age at onset of spectacle use was 5 years of age for both phenotypes. Information on self-reported visual symptoms was not available in seven patients. Symptoms of blurred vision due to early CRD and myopia often conflate, and these findings are confounded by the patient's inability to distinguish between the two in retrospect.

**Figure 2:**
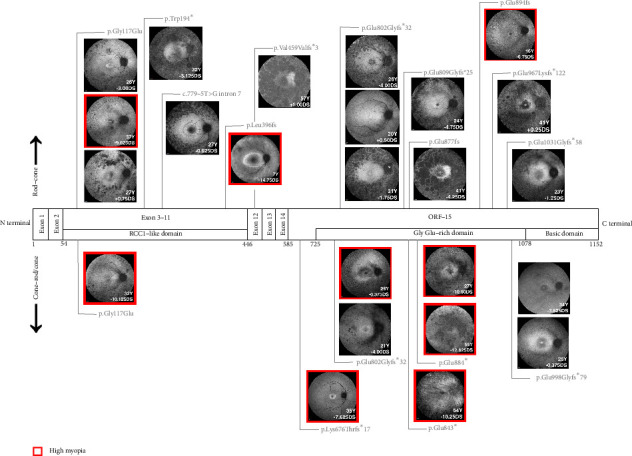
A schematic structure of the RPGR^ORF15^ isoform with the right eye fundus autofluorescence images from 24 male patients overlain according to pathogenic variant location. The structure was not drawn to scale. Denoted on each image is the patient age at the time of refraction, best vision sphere for the right eye and RPGR pathogenic mutation at the protein level. Images with a red border indicate patients with high myopia; dioptrically defined as > −6.00DS. Distinct phenotype categories were identified in this cohort: Rod-cone phenotype plotted above the gene structure and cone-rod/cone-only below. Note the preponderance of high myopia in the cone-rod/cone-only phenotype.

The protein change for each patient is shown diagrammatically in Figure 2, where the autofluorescence images from all patients are plotted according to pathogenic variant location in *RPGR* gene. Frameshift mutations were the most frequent type of mutation found in this patient cohort (16/24, 63%) followed by missense mutations (4/24, 16%) and nonsense mutations (4/24, 16%). A single intronic splice defect was identified in patient 6. The nonsense mutation c.2650G>T, p.Glu884^∗^ was most frequently featured (3/9, 33%) among patients with high myopia.

A key trend in this cohort was that patients with a cone-rod/cone-only phenotype were associated with high myopia, although notable outliers to this trend were identified and are outlined herein. Patient 5 presented at the age of 25 with a low myopic refractive error and was classified with a late-onset, cone-rod phenotype, reportedly noticing dyschromatopsia and nyctalopia in his teenage years. Disease progression rate was estimated by an ophthalmologist based on a combination of the patient's age and clinical picture at presentation, retrospective analysis of multimodal imaging, microperimetry and patient history. Conversely, patient 19 presented at the age of 37 with a severe rod-cone phenotype and reduced BCVA, demonstrating a faster clinically determined disease progression, accompanied by high myopia of around −9.50DS. Patients 17 and 24 were also classified with an early-onset, fast-progressing disease. They presented with severe rod-cone retinal dystrophy and established cone involvement at a relatively young age (16 and 7 years old, respectively). Similar to patient 19, they both exhibited high myopia of −6.75DS and −14.75DS, respectively.

## 4. Discussion

Associations between cone photoreceptor dysfunction and myopia have previously been identified [13, 17]. We report a novel aspect of this observation: specifically, that cone photoreceptor dysfunction occurring during early childhood is more strongly associated with high myopia in patients with *RPGR*-related retinal dystrophy. This study contributes knowledge that the period of onset of cone photoreceptor degeneration is key to the development of high myopia and highlights the importance of the early onset of this retinal degeneration.

In accordance with the established natural history of CRDs [7], it is expected that patients with a cone-rod phenotype are affected by earlier dysfunction of cone photoreceptors compared to those with a rod-cone phenotype. The cone-only phenotype tends to have a more delayed onset with slower progression to central macular atrophy compared to the cone-rod phenotype [7]. Notable outliers were described in detail (patients 5, 17, 19, and 24) and serve as figurative controls for our observations. They represent a departure from the general trend seen in this cohort which can be accounted for by the onset and progression rate of their retinal dystrophy. In this way, we provide clinical evidence to suggest that cone photoreceptor degeneration occurring in early childhood, as opposed to later, is associated with high myopia.

It is possible that these outlier patients deviated from the general trend in this cohort due to other uncontrolled factors in this analysis. For example, a patient's baseline genetic predisposition to myopia (influenced by familial myopia and specific gene variations linked with myopia) and environmental modifiers such as educational level and outdoor time.

The mechanism by which cone dysfunction leads to high myopia is unknown, but several theories exist. Image degradation caused by cone dysfunction can act as a stimulus to increase axial length, particularly if present during the critical development period before around age 7 years [18]. During this period, the visual experience is fundamental in coordinating eye growth to align the eye's refractive system to its axial length in emmetropization [19]. Mechanistically, it has been proposed that an increase in the level of scleral proteinases may modify the biomechanical properties of the sclera by contributing to tissue remodeling and growth [20]. This process has been proven in animal models of chicks and monkeys which demonstrated increased ocular length after retinal image degradation [21, 22]. In cone-dominated retinal dystrophies, the image degradation would be experienced maximally within the perifoveal region, as reflected by the topographical distribution of cones in the retina [23]. Thus, the distribution of cones may indicate a key role for the fovea or perifoveal region in the development of high myopia. The term “image degradation” is designated to several optical properties: optical defocus, alterations to perceived light intensity and light wavelength can all cause image degradation, which may have consequences for ocular growth [24].

Cone-rod retinal dystrophies often initially present with reduced macular sensitivity which may alter the effective light intensity experienced by the retina and disturb visual input to the visual cortex. This theory concurs with genome-wide pathway analyses that have found light signaling and processing pathways in the retina to be important in initiating myopia [25]. Whole transcriptome meta-analysis in animal models of complete congenital stationary night blindness, another inherited retinal disease, similarly found that abnormalities in the light-dependent retinal signaling cascade were linked to myopia [26]. Moreover, the aberrant spectral composition of light perceived by the retina as a result of degenerated cones, compounded with the expected longitudinal chromatic aberration experienced by the eye, has also been suggested as a signaling cue for scleral tissue remodeling and growth [19].

The selective, initial degeneration of either cone or rod photoreceptors in *RPGR*-related retinal dystrophy can inadvertently act as a model to predict how these separate photoreceptor classes contribute to refractive error development. In the outlier patients identified, differences in the onset of cone photoreceptor dysfunction were a key differentiator in the outcome of myopia severity. Although patients 17, 19, and 24 were classified with a rod-cone phenotype, their fast-progressing disease likely caused cone involvement early in life which may have contributed to their highly myopic refractive error. Patient 5 was clinically classified with a late-onset cone-rod phenotype, with cone dysfunction that likely occurred beyond the formative early childhood years. It is reasonable to suggest that if cones degenerate from birth or early childhood, there is a predisposition to high myopia.

The hypothesis posed by our study can be generalized to other early-onset, cone-dominated dystrophies associated with myopia, such as *CDHR1* and *RP2* retinal dystrophies [5, 14, 27]. Uniquely, RPGR is a ciliary protein with a dual role in photoreceptor structure (via cilia) and light signaling pathways (e.g., dopamine metabolism) which may synergistically drive myopia. Centrosomal protein 290 (CEP290) localizes to the connecting cilium of photoreceptors and interacts with several microtubule-based transport proteins, including RPGR. In *CEP290*-associated Leber's congenital amaurosis, early disease stages are characterized by relative preservation of the central, cone-rich retina, and high hyperopia [28]. The rarer, cone-dominated *CEP290*-associated dystrophy has been found to present with myopia [29, 30]. Thus, our observation that early-onset cone degeneration is associated with high myopia is also seen in CEP290; another ciliary protein which shares a similar structural and functional role with RPGR.

Mutations in cone-specific phototransduction genes not associated with ciliopathies—for example, genes causing the early-onset achromatopsia—may induce myopia solely via image degradation mechanisms mediated by dysfunctional cone signaling. These mutations disrupt the highly regulated kinetics of the phototransduction cascade, which govern visual sensitivity [31]. For instance, *PDE6C*-associated achromatopsia affects the phosphodiesterase 6 (PDE6) protein involved in cone phototransduction and frequently presents with high myopia and macular atrophy [32]. *GNAT2*, encoding the α-subunit of cone transduction, causes achromatopsia with relatively preserved cone mosaic and structure on retinal imaging and presents with variable refractive error [33]. Mutations in *CNGA3* and *CNGB3* cause achromatopsia but do not show a consistent association with myopia [34]. Blue cone monochromacy is characterized by attenuated or absent L-opsin and M-opsin expression encoded by *OPN1LW* and *OPN1MW*, and is strongly linked to high myopia [35]. Termination of cone opsin activity is regulated by cone arrestin, encoded by *ARR3* in M/L cones, and *ARR3* mutations are linked to early-onset high myopia [36]. The broad spectrum of refractive error described here implies that early-onset dysfunction in cone phototransduction partly, but not wholly, stimulates myopia development. Further research is required to comprehend the complex molecular mechanisms and pathogenesis of myopia in *RPGR*-related retinal dystrophy.

### 4.1. Treatment and Management of High Myopia in *RPGR*-Related Retinal Dystrophy

There is little to no evidence base surrounding the use of myopia control treatments in patients with retinal dystrophies. The rarity of these diseases can make it difficult to gather the minimum patient number needed in power analyses to determine a treatment effect. In the general population, increasing outdoor time has been recognized as a promising intervention method in slowing myopia progression but is of limited prescriptive value for patients in whom degenerated photoreceptors have a reduced capacity to absorb and process light. Moreover, many spectacle or soft contact lens treatments induce peripheral blur which may not be efficacious due to the presence of peripheral retinal degeneration in patients with *RPGR*-related retinal dystrophy. Success with orthokeratology myopia control treatments may be tempered by corneal topography requirements and the risk of microbial keratitis infection inherent in contact lens wear.

Pharmacological treatment may be a more successful option. A recent study found that high-dose atropine effectively reduced myopia progression in children with a strong monogenic driver, although axial elongation was notably harder to control in those with mutations in *RPGR* [37]. The presumed site of action of atropine is downstream of retinal signaling, at the level of the choroid which may account for the promising treatment effects reported to date. The use of high dose atropine (0.5%) has been used in myopic children of preschool age due to their greater lifetime risk of myopia progression, and this may be better tolerated due to the high levels of accommodative reserve present at this age. Clinicians that offer treatment for myopia control in inherited retinal diseases do so largely from professional experience, and not evidence-based studies in humans, as patients with retinal dystrophy are often excluded from recruitment into major clinical trials in myopia. It is important to consider that photophobia and glare are commonly reported symptoms in the cone-rod phenotype of *RPGR*-related retinal dystrophy and so, the long-term prescription of pupil dilating eye drops in these patients may be contraindicated or complicated by nonadherence.

The sequelae of structural complications from pathological myopia are likely to have a more profound effect on patients with retinal dystrophy than those without. For example, consider the risk that myopic maculopathy may pose on the islands of preserved retina that remain in patients with end-stage *RPGR*-related retinal dystrophy. If high myopia is linked to a faster decline in BCVA [12], then the weight of clinical evidence may prompt clinicians to consider atropine treatment. High myopia will also have implications for inclusion into gene therapy clinical trials which may adopt stricter criteria to exclude patients with a more aggressive clinical course and who may not demonstrate a detectable treatment effect.

Insight into the mechanisms of myopia development in this cohort may help to inform clinicians on the optimal age to treat as more therapies are approved. The added risk of developing high myopia in the cone-rod phenotype of *RPGR*-related retinal dystrophy, on balance with the inherent risk of retinal surgery, may be an early intervention factor to treat patients with gene therapy sooner. Thus, understanding subtle phenotypic variations can aid clinicians in prioritizing patients and determining optimal treatment windows when prescribing novel therapies that are in development.

### 4.2. Limitations

The study data does not capture the rate or presence of myopia progression; future studies evaluating refractive error would benefit from a longitudinal study design with serial measurements and patient follow-up. Moreover, other established risk factors that can affect myopia development were not accounted for, to include educational level and outdoor time during early childhood and familial myopia.

The retrospective nature of this case-series study meant that the estimations of cone involvement and the onset of cone photoreceptor degeneration were based on historical data, rather than direct observation. Where data were self-reported by patients, recall bias could have affected the accuracy of these estimates.

## 5. Conclusion

Our study of patients with *RPGR*-related retinal dystrophy suggests an association between early-onset cone photoreceptor degeneration and high myopia. Image degradation primarily due to cone photoreceptor dysfunction may act as a stimulus to drive high myopia development in early childhood. Better understanding into the mechanisms of myopia development via further controlled studies can prompt earlier intervention strategies to mitigate disease in affected patients and minimize risks of retinal gene therapy surgery for patients enrolling in clinical trials.

## Figures and Tables

**Table 1:** Patient demographic characteristics and clinical information.

Case number	Age^∗^ (years)	Self-reported age of onset and initial symptom related to retinal dystrophy (years)	Self-reported age onset of spectacle use (years)	BCVA (letter score) Right/left	Refractive error^†^ (DS) Right/left	Axial length (mm) Right/left	Clinical phenotype
1	41			35/60	+0.25/+0.50		Rod-cone
2	20	8/nyctalopia	8	81/75	+0.50/+0.625	21.57/21.78	Rod-cone
3	27	0	Not worn	65/69	+0.75/+0.75	21.57/21.36	Rod-cone
4	57			16/19	+1.00/+0.625		Rod-cone
**5**	**25**	**13/dyschromatopsia and nyctalopia**	**23**	**74/71**	**−0.375/−0.75**	**22.54/22.39**	**Cone-rod**
6	27			82/80	−0.625/−0.75		Rod-cone
7	23			63/63	−1.25/−5.00		Rod-cone
8	31	17/nyctalopia	13	72/74	−1.75/−2.375	22.97/22.99	Rod-cone
9	26			74/74	−3.00/−3.25	23.96/23.87	Rod-cone
10	32	4/visual field restriction	4	63/67	−3.125/−4.125	24.16/24.07	Rod-cone
**11**	**34**	**15/nyctalopia**		**47/29**	**−3.625/−1.25**	**24.12/23.99**	**Cone-rod**
**12**	**21**	**10/nyctalopia**		**66/68**	**−4.00/−3.125**		**Cone-rod**
13	26			35/35	−4.00/−4.00		Rod-cone
14	41	9/visual field restriction and nyctalopia	5	20/56	−4.25/−3.75	25.16/24.77	Rod-cone
15	24	15/visual field restriction and nyctalopia	5	75/74	−4.75/−3.875	24.46/24.03	Rod-cone
**16**	**25**	**20/nyctalopia**	**3**	**73/75**	**−6.375/−5.75**	**26.69/26.45**	**Cone-rod**
17	16	5/nyctalopia		65/75	−6.75/−5.75		Rod-cone
**18**	**35**	**29**	**7**	**55/60**	**−7.625/−7.50**		**Cone**
19	37	13/nyctalopia	0	64/68	−9.625/−9.25	26.81/26.92	Rod-cone
**20**	**32**	**25/nyctalopia**	**5**	**57/59**	**−10.125/−9.50**	**25.0/24.87**	**Cone-rod**
**21**	**54**			**60/20**	**−10.25/−12.00**		**Cone-rod**
**22**	**27**	**15/nyctalopia**	**3**	**66/63**	**−10.50/−10.875**	**27.89/27.85**	**Cone-rod**
**23**	**55**	**0/nyctalopia**	**5**	**35/41**	**−12.625/−12.25**	**26.21/26.24**	**Cone-rod**
24	7	2/visual field restriction and nyctalopia	7	70/80	−14.75/−14.50		Rod-cone

*Note:* Bold indicate patients displaying a cone-rod/cone-only phenotype. Age 0 indicates patients who have known of visual impairment as far as they could remember. Information on self-reported visual symptoms spectacle use was not available in Patients 1, 4, 6, 7, 9, 13, and 21. Measurements of axial length were not available in Patients 1, 4, 6, 7, 12, 13, 17, 18, 21, and 24.

^∗^Age at refraction.

^†^Refractive error recorded as best vision sphere (sphere power plus half of the cylinder power) calculated to three decimal places.

## Data Availability

The data that support the findings of this study are available from the corresponding author upon reasonable request.
